# Data integration by fuzzy similarity-based hierarchical clustering

**DOI:** 10.1186/s12859-020-03567-6

**Published:** 2020-08-21

**Authors:** Angelo Ciaramella, Davide Nardone, Antonino Staiano

**Affiliations:** 1 Dipartimento di Scienze e Tecnologie, Università degli Studi di Napoli “Parthenope”, Centro Direzionale, C4 Island, Naples, 80143 Italy; 2 Hitachi Rail STS, Via Argine, 425, Naples, 80147 Italy

**Keywords:** Multi-omics data, Data integration, Hierarchical clustering, Fuzzy similarity, Fuzzy aggregation

## Abstract

**Background:**

High throughput methods, in biological and biomedical fields, acquire a large number of molecular parameters or omics data by a single experiment. Combining these omics data can significantly increase the capability for recovering fine-tuned structures or reducing the effects of experimental and biological noise in data.

**Results:**

In this work we propose a multi-view integration methodology (named *FH*-Clust) for identifying patient subgroups from different *omics* information (e.g., *Gene Expression*, *Mirna Expression*, *Methylation*). In particular, hierarchical structures of patient data are obtained in each omic (or view) and finally their topologies are merged by consensus matrix. One of the main aspects of this methodology, is the use of a measure of dissimilarity between sets of observations, by using an appropriate metric. For each view, a dendrogram is obtained by using a hierarchical clustering based on a fuzzy equivalence relation with *Łukasiewicz* valued fuzzy similarity. Finally, a consensus matrix, that is a representative information of all dendrograms, is formed by combining multiple hierarchical agglomerations by an approach based on transitive consensus matrix construction. Several experiments and comparisons are made on real data (e.g., Glioblastoma, Prostate Cancer) to assess the proposed approach.

**Conclusions:**

Fuzzy logic allows us to introduce more flexible data agglomeration techniques. From the analysis of scientific literature, it appears to be the first time that a model based on fuzzy logic is used for the agglomeration of multi-omic data. The results suggest that *FH*-Clust provides better prognostic value and clinical significance compared to the analysis of single-omic data alone and it is very competitive with respect to other techniques from literature.

## Background

Nowadays, high throughput methods, in biological and biomedical fields, acquire a large number of molecular parameters by a single experiment [1]. In particular, such measured parameters are collected in “omics” datasets (e.g., genomics, transcriptomics, methylomics). Among multiple measured parameters, DNA genome sequence, RNA expression and DNA methylation are representative instances. For individually analysing such data, several methodologies have been introduced in literature, even though, recently, a number of studies pointed out the best performance coming from the integration of multi-omics data. For instance, analysing each omic (or *view* in the machine learning jargon), set separately, fundamental patterns can be detected from data, however some fine-tuned structures, such as cancer sub-types, can be highlighted by both gene expression and DNA methylation information, so that multi-omics analysis can reduce the effects of experimental and biological noise in data [2]. From literature, three kinds of integration methodologies emerge:

- **early integration**, builds a single feature-based matrix by concatenating each omic dataset (i.e., *view*) and applies a single-omic analysis;
- **intermediate integration**, builds a joint representation of data given the views;
- **late integration**, each omic is analysed separately and the solutions are integrated.

**Fig. 1:**
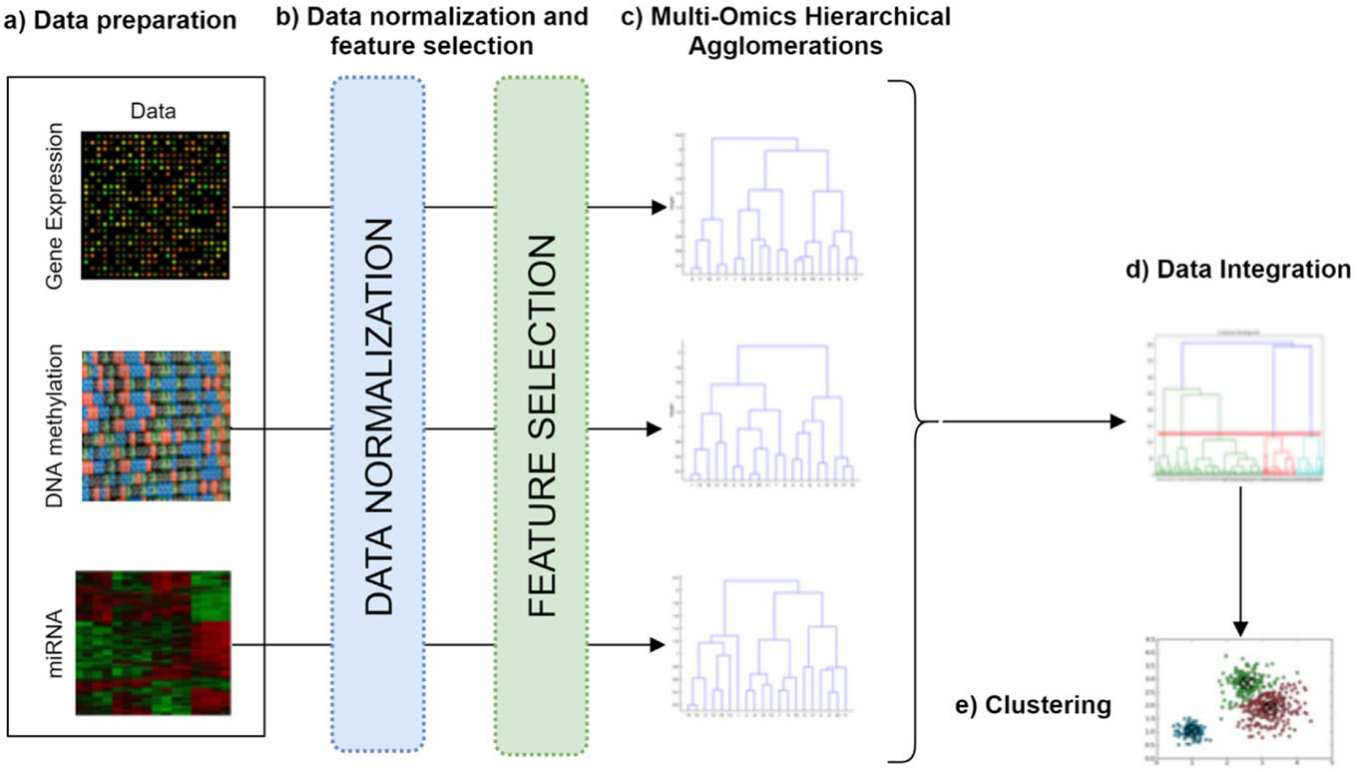
Proposed approach: **a** Data preparation; **b** Data normalization and feature selection; **c** Multi-omics hierarchical agglomerations; **d** Data integration; **e** clustering and visualization

In general, late integration methods, and in particular clustering, are preferred when the analysis combines continuous and discrete data together. For a review of integration approaches and their comparisons, the reader may refer to [3]. In this work, a multi-view clustering methodology, named *FH*-Clust, is introduced (see Fig. 1 for its general schema) for identifying patient subgroups from different omics information or datasets (e.g., Gene Expression, Mirna Expression, Methylation). Specifically, for each omic dataset a fuzzy-based hierarchical clustering approach is adopted and finally the results are merged by consensus matrix. The idea behind the proposed approach comes from observing that a hierarchical clustering dendrogram can be associated with a *Łukasiewicz* fuzzy similarity-based equivalence relation, so that a consensus matrix, that is the representative information of all dendrograms, is derived by combining multiple hierarchical agglomerations following an approach based on transitive consensus matrix construction.

## Methods

Cluster analysis or clustering is an unsupervised technique that aims at agglomerating a set of patterns in homogeneous groups or clusters [4, 5]. Hierarchical Clustering (HC) is one of several different available techniques for clustering which seeks to build a hierarchy of clusters, and it can be of two types, namely *agglomerative*, where each sample starts in its own cluster, and pairs of clusters are merged as one moves up the hierarchy, or *divisive*, where all samples start in one cluster, and splits are performed recursively as one moves down the hierarchy [6]. Thus, HC aims at grouping similar objects into a cluster, and were the endpoint is a set of clusters where each cluster is distinct from each other, and the objects within each cluster are broadly similar to each other. HC can be performed either on a distance matrix or raw data. Agglomerative HC starts by treating each observation as a separate cluster, and it repeatedly executes the following two steps: (1) identifies the two clusters that are closest together, and (2) merges the two most similar clusters. This process continues until all the clusters are merged together.

The main output of HC is a dendrogram, which shows the hierarchical relationship between the clusters distances. Many distance metrics have been developed and the choice should be made based on theoretical concerns from the domain of study.

Later on, it is necessary to determine how the distance is computed (e.g., single-linkage, complete-linkage, average-linkage). As with distance metrics, the choice of linkage criteria should be based on theoretical considerations from the application domain.

**Fig. 2:**
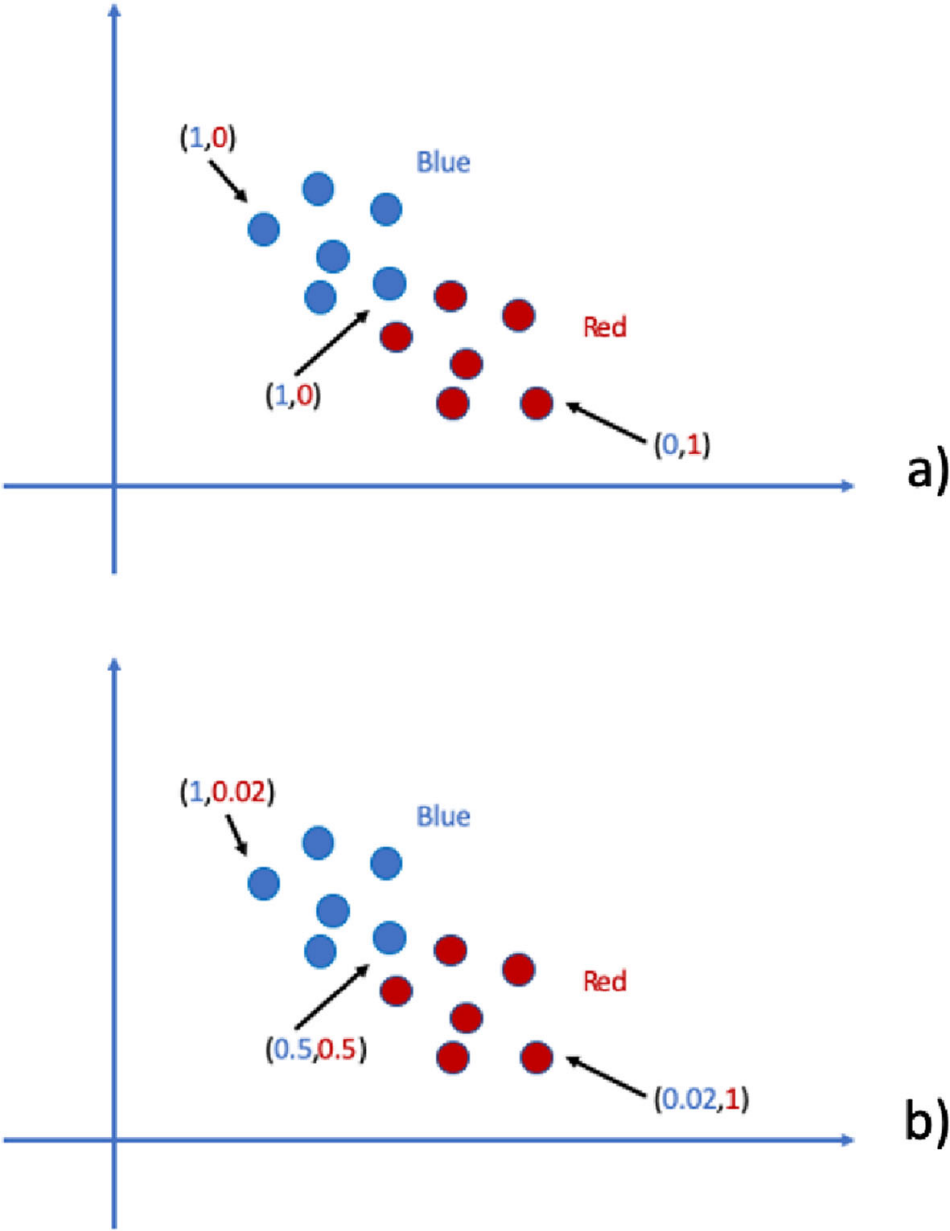
Hard vs Fuzzy in symptom risk example: **a** Hard categorization; **b** Fuzzy categorization
- Computation of a fuzzy similarity matrix (or dendrogram) for all models, at a given time;
- Construction of a consensus matrix for all hierarchical agglomerations.

In non-fuzzy clustering (or hard clustering) data is divided into distinct clusters and each data point can only belong to exactly one cluster. In fuzzy clustering, data points can potentially belong to multiple clusters. For example, in hard clustering, given some parameters, a “symptom” can be (in a mutually exclusive way) present or absent (red or blue) whereas, in fuzzy clustering, that “symptom” could (simultaneously) be of some grade red *and* some other grade blue. In Fig. 2, a comparison between hard and fuzzy categorisation is shown. The reader can refer to [7] for a recent comparison between hard and fuzzy clustering. In this work, we introduce a data integration methodology based on fuzzy concepts. In particular, we associate a dendrogram to a fuzzy equivalence relation (i.e., *Łukasiewicz* valued fuzzy similarity), so that a consensus matrix in a multi-view clustering, that is the representative information of all dendrograms, can be obtained from multiple hierarchical agglomerations [8, 9]. The main steps of fuzzy agglomeration can be summarised as follows:

- Characterisation of membership functions;

### Membership functions

When dealing with clustering tasks, Fuzzy Logic (FL) permits to obtain a *soft* clustering instead of an *hard* clustering of data [10]. Specifically, data points can belong to more than one cluster simultaneously. The fundamental concept in FL, upon which all the subsequent theory is constructed, is the notion of fuzzy set, a generalisation of a crisp set from classical set theory.

A fuzzy set generalises a crisp set by allowing its characteristic function, i.e., its membership function, assuming values in the interval [0,1] rather than in the set {0,1}. In this way, a given item belongs to the fuzzy set with a degree of truth ranging from *do not belong at all* (i.e., its membership function assumes value 0) to *completely belong* (i.e., the membership function assumes value 1). In FL applications, fuzzy sets make it possible to represent qualitative (non-numeric) values (i.e., linguistic variables such as *High*, *Medium*, *Low*) for approximate reasoning, inference or fuzzy control systems. Linguistic variables can be represented by fuzzy sets through a transformation step called fuzzification, and it is achieved by using different types of membership functions representing the degree of truth to which a given input sample belongs to a fuzzy set (see “Membership Functions” section in Supplementary Material).

### Fuzzy similarity matrix

A measure of similarity or dissimilarity defines the resemblance between two samples or objects. Similarity measure is a significant means for measuring uncertain information. Fuzzy similarity measure is a measure that depicts the closeness among fuzzy sets and has been used for dealing issues of pattern recognition and clustering analysis.

A binary fuzzy relation that is reflexive, symmetric, and transitive is known as a similarity relation. Fuzzy similarity relations are the generalisation of equivalence relations, in binary crisp relations, to binary fuzzy relations. In details, a fuzzy similarity relation can be considered to effectively group elements into crisp sets whose members are similar to each other to some specified grade and it is a generalization of classical equivalence relation as described in “Fuzzy Similarity” section in Supplementary Material. In order to introduce the fuzzy similarity, in the following, we focus on the properties of the Łukasiewicz *t*-norm (*t*_**L**_) and the *bi-residuum*. In this way we obtain a fuzzy equivalence relation that can be used for building dendrogram. For more details in the derivation of these results see “Fuzzy Similarity” section in Supplementary Material.

### Dendrogram and consensus matrix

If a similarity relation is *min-transitive* (i.e., *t*= min) then it implies the existence of the dendrogram (see “Dendrogram and Consensus Matrix” section in SupplementaryMaterial for details). The min-transitive closure of a relation matrix *R* can be easily computed and the overall process is described in Algorithm 1.

The last ingredient to accomplish an agglomerative clustering is a dissimilarity relation. Here we considered the following result [11]:

#### **Lemma 1**

Letting *R* be a similarity relation with the elements *R*〈*x*,*y*〉∈[0,1] and letting *D* be a dissimilarity relation, which is obtained from *R* by

1 $$ D(x,y) = 1 - R \langle x,y \rangle  $$

then *D* is ultrametric iif *R* is min-transitive.

**Fig. 3:**
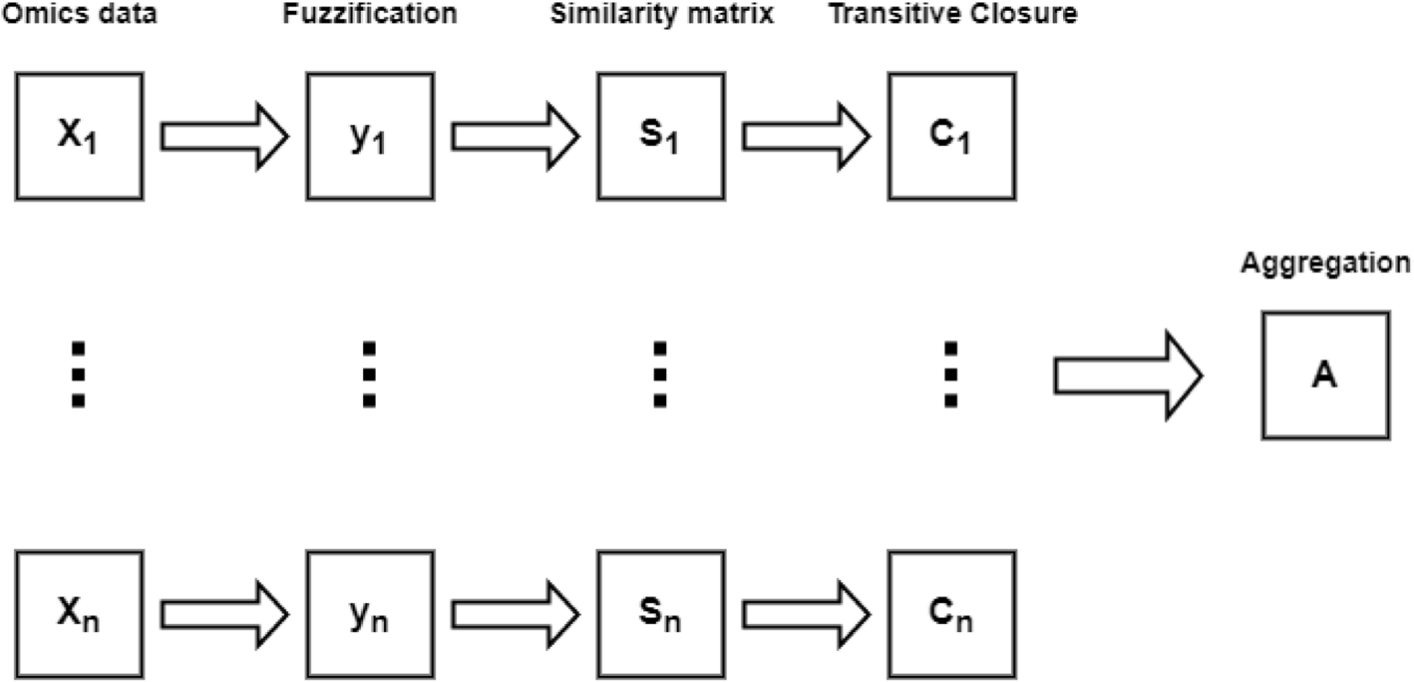
Workflow of the fuzzy based hierarchical clustering

In other words, we have a one-to-one correspondence between min-transitive similarity matrices and dendrogram and between ultrametric dissimilarity matrices and dendrograms. Finally, after the dendrograms have been obtained each time, a consensus matrix, i.e., the representative information of all dendrograms is obtained by combining the transitive closures (i.e., max-min operation) [11]. The overall approach is described in Algorithm 2. The overall workflow of the proposed approach is summarised in Fig. 3. In particular, for each omic data set **X**_*i*_ a fuzzification step is adopted for obtaining the new data set **Y**_*i*_ (see Supplementary Material). Successively, adopting a fuzzy similarity measure the similarity matrix **S**_*i*_ is computed and to guarantee the transitive closure of the matrix a new matrix **C**_*i*_ is computed (see Algorithm 1). Finally, all the **C**_*i*_ matrices are collected for obtaining the consensus matrix **A** and the overall final dendrogram (see Algorithm 2).

**Fig. 4:**
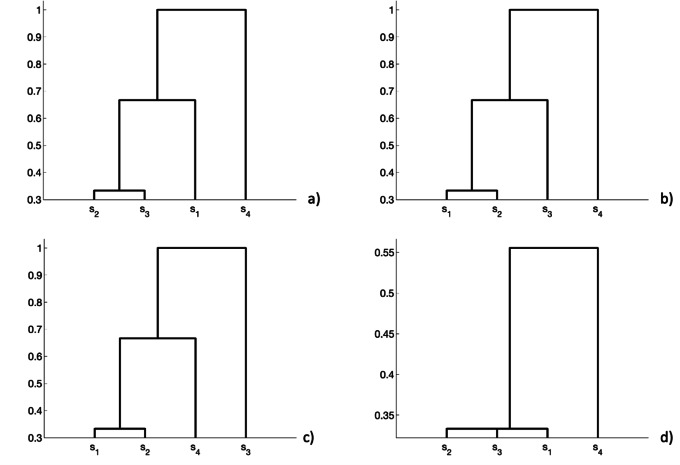
Combination algorithm: **a**-**b**-**c** input dendrograms; **d** combined hierarchy

**Fig. 5:**
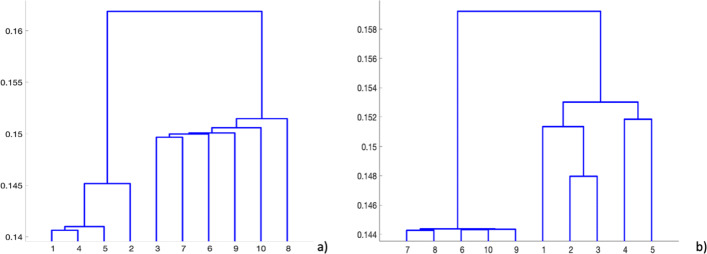
Crisp Hierarchical Clustering vs Fuzzy based Hierarchical Clustering: **a** dendrogram of Euclidean based Hierchical Clustering; **b** dendrogram of similarity based Hierachical Clustering


In Fig. 4, we show an example that summarize a realistic agglomeration result. We plot in Figs. 4a-b-c three input hierarchies obtained on datasets that should be combined. In this case, four sequences of patients are considered, namely *s*_1_,*s*_2_,*s*_3_ and *s*_4_, respectively. In Fig. 4d, we show the final result by agglomerating dendrograms. We observe that the output hierarchy contains clusters (*s*_1_,*s*_2_,*s*_3_) and (*s*_1_,*s*_2_,*s*_3_,*s*_4_) at different levels and each of these clusters (e.g., (*s*_1_,*s*_2_,*s*_3_)) are repeated at least in two out of the three input dendrograms. Moreover, it is worth stressing that the proposed approach, based on the agglomeration of dendrograms, can also be applied with commonly used metrics (e.g., Euclidean distance). In Fig. 5, we show a comparison between the dendrograms obtained by using an Euclidean metric and a similarity based approach (i.e., Łukasiewicz *t*-norm), respectively. In this realistic example, we simulate three omic data sets with 10 rows (i.e., number of patients) and 100 columns (i.e., features). We split the single datasets in two partitions (or clusters) such that the first 5 rows are random samples from a standard normal distribution with variance 1 and the other 5 rows have the same distribution with variance 0.5, obtaining a sort of an overlap. We observe that both methods find two separated clusters, but the similarity based approach in Fig. 5b, permits to obtain a perfect separation of the source partitions.

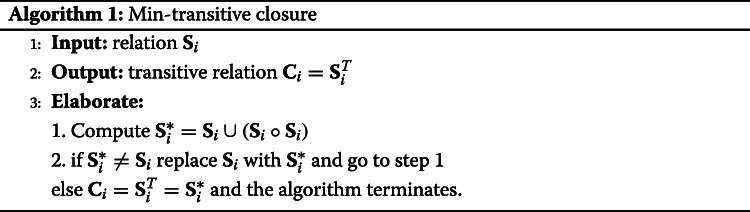


## Results and discussion

In the following we describe the behaviour of the proposed methodology on multi-omics benchmark datasets.


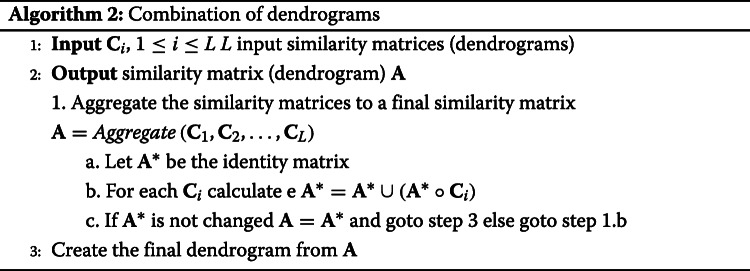


### Omics datasets

We consider 10 multi-omics cancer datasets available from The Cancer Genome Atlas (TCGA) [3]. TCGA is a large multi-omic repository of data on thousands of cancer patients. All datasets contain three omics: gene expression, miRNA expression and DNA methylation^1^. In Table 1 are summarised the main properties of the datasets, namely, *Acute Myeloid Leukemia* (AML), *Breast Invasive Carcinoma* (BIC), *Colon Adenocarcinoma* (COAD), *Glioblastoma Multiforme* (GBM), *Kidney Renal Clear Cell Carcinoma* (KIRC), *Liver Hepatocellular Carcinoma* (LIHC), *Lung Squamous Cell Carcinoma* (LUSC), *Skim Cutaneous Melanoma* (SKCM), *Ovarian serous cystadenocarcinoma* (OV), *Sarcoma* (SARC). The number of patients ranges from 170 for AML to 621 for BIC.

**Table 1:** Datasets description: Three omics are provided for each dataset, respectively DNA gene expression, miRNA and Methylation

	#Cases	DNA			miRNA			Methy			Multi-Omics		
Dataset	-	*ORI*	*LN*	*RF*	*ORI*	*LN*	*RF*	*ORI*	*LN*	*RF*	*ORI*	*LN*	*RF*
AML	170	20531	2000	1997	5000	2000	1999	705	558	553	26236	4558	4529
BIC	621	20531	2000	2000	5000	2000	2000	1046	891	854	26577	4891	4854
COAD	220	20531	2000	2000	5000	2000	2000	705	613	591	26236	4613	4590
GBM	274	12042	2000	2000	5000	2000	2000	534	534	534	17576	4534	4534
KIRC	183	20531	2000	1999	5000	2000	1999	1046	796	754	26577	4796	4752
LIHC	367	20531	2000	2000	5000	2000	2000	1046	852	826	26577	4852	4366
LUSC	341	20531	2000	2000	5000	2000	2000	1046	878	850	26577	4878	4850
SKCM	448	20531	2000	2000	5000	2000	2000	1046	901	874	26577	4901	4874
OV	287	20531	2000	2000	5000	2000	2000	705	616	600	26236	4616	4600
SARC	257	20531	2000	2000	5000	2000	2000	1046	838	805	26577	4838	4805

The number of features at each variable selection method is shown. ORI: Original variable dimension, LN: Logarithm and normalisation and, RF: Random Forest based on Mean Decrease Gini index

### Multi-view clustering algorithms

For validating the effectiveness of our model, we compared it against several categories of multi-view clustering algorithms^2^:

- K-means and Spectral Clustering techniques [3];
- LRACluster [12]: It is a low-rank approximation based integrative probabilistic model to fast find the shared principal subspace across multiple data types;
- PINS [13]: Perturbation clustering for data integration and disease subtyping (PINS) is able to address subtype discovery, as well as integration of multiple data types. The algorithm is built upon the resilience of patient connectivity and cluster ensembles to ensure robustness against noise and bias;
- SNF [14]: Similarity network fusion (SNF) allows for discovery of disease subtypes through integration of several types of high-throughput data on a genomic scale. SNF creates a fused network of patients using a metric fusion technique and then partitions the data using spectral clustering. SNF appears to be the state of the art in this area and has proven to be very powerful. However, the unstable nature of kernel-based clustering makes the algorithm sensitive to small changes in molecular measurements or in its parameter settings.
- MCCA [15]: Multi Canonical Correlation Analysis (MCCA), which extends the application of Canonical Correlation Analysis (CCA) to more than two views, is one of the most widely used dimension reduction method for finding linear relations between two or more multidimensional random variables.

### Evaluation metrics

In order to assess the performance of each method, we adopt three evaluation metrics that are: the *logrank test*, the *enrichment* of clinical labels in the clusters and the methods runtime [3]. The *logrank test* assumes that if clusters of patients have significantly different survival, they are different in a biologically meaningful way. For the *enrichment* of clinical labels in clusters, six clinical labels are considered: gender, age at diagnosis, pathologic tumor, pathologic metastases, pathologic lymph nodes and pathologic stage. The four latter parameters are discrete pathological parameters, measuring the progression of the tumor, metastases and cancer in lymph nodes, and the total progression (pathologic stage). Enrichment for discrete parameters was calculated using the *χ*^2^ test for independence, and for numeric parameters using Kruskal-Wallis test. Not all clinical parameters were available for all cancer types, so a total of 41 clinical parameters were available for testing. To derive a *p*-value for the logrank test, the *χ*^2^ test for independence, the Kruskal-Wallis test and the statistic for these three tests is assumed to have *χ*^2^ distribution [3].

### Pre-processing

TCGA datasets were preprocessed as follows: patients and features with more than 20% missing values were removed, and missing values were imputed using k-nearest neighbor imputation. The sequence features were log-transformed. The 2000 features with highest variance from gene-expression and methylation omics were selected. In the miRNA omic, features with zero variance were filtered. All features were then normalized to have zero mean and standard deviation 1. For methylation, we selected the 5000 features with maximal variance in each dataset and also adopted the standard pipeline proposed in [16], whose procedure filters out the probes from the X and Y chromosomes or probes that are known to have common SNPs at the CpG site.

A further unsupervised variable selection step has been performed by using the *Mean Decrease Gini* [17] based on Random Forest [18]. The Mean Decrease in Gini is the average of a variable total decrease in node impurity, weighted by the proportion of samples reaching that node in each individual decision tree in the forest. This is effectively a measure of how important a variable is for estimating the value of the target variable across all of the trees that make up the forest. A higher Mean Decrease in Gini indicates higher variable importance, therefore the most important variables to the model is the highest in the plot with the largest Mean Decrease in Gini Values, conversely, the least important variable is the lowest in the plot with the smallest Mean Decrease in Gini values. By following this strategy, we cut-off all those variables whose importance is zero. The number of variable cut-off at each step is summarised in Table 1.

### Experimental results

In the experiments, for all methods, the number of searched clusters is selected in the range [2−15]. To determine the number of clusters for a method we used the “elbow method”. To automatically pick out the optimal elbow rather than choose it manually, we used as approximation the second derivative of a vector **v**

2 $$  \begin{aligned} {\mathbf v}\left[i+1\right] + {\mathbf v}\left[i-1\right] - 2{\mathbf v}[i]. \end{aligned}  $$

In particular, we consider the index *i* that brings this expression to a maximum or minimum (depending on whether **v** increases or decreases). For all methods, we adhered to the guidelines for usage and parameter selection given by the developers. In some cases, where no information was provided by the authors, we devised parameter selection methods. We performed the same process pipeline used in [3] for evaluating the performance of our method. All methods were run on a 24 multi-core Intel(R) Xeon(R) CPU E5-2620 v3 @ 2.40GHz with 64 GB RAM. In the following, the obtained experimental results are described.

**Fig. 6:**
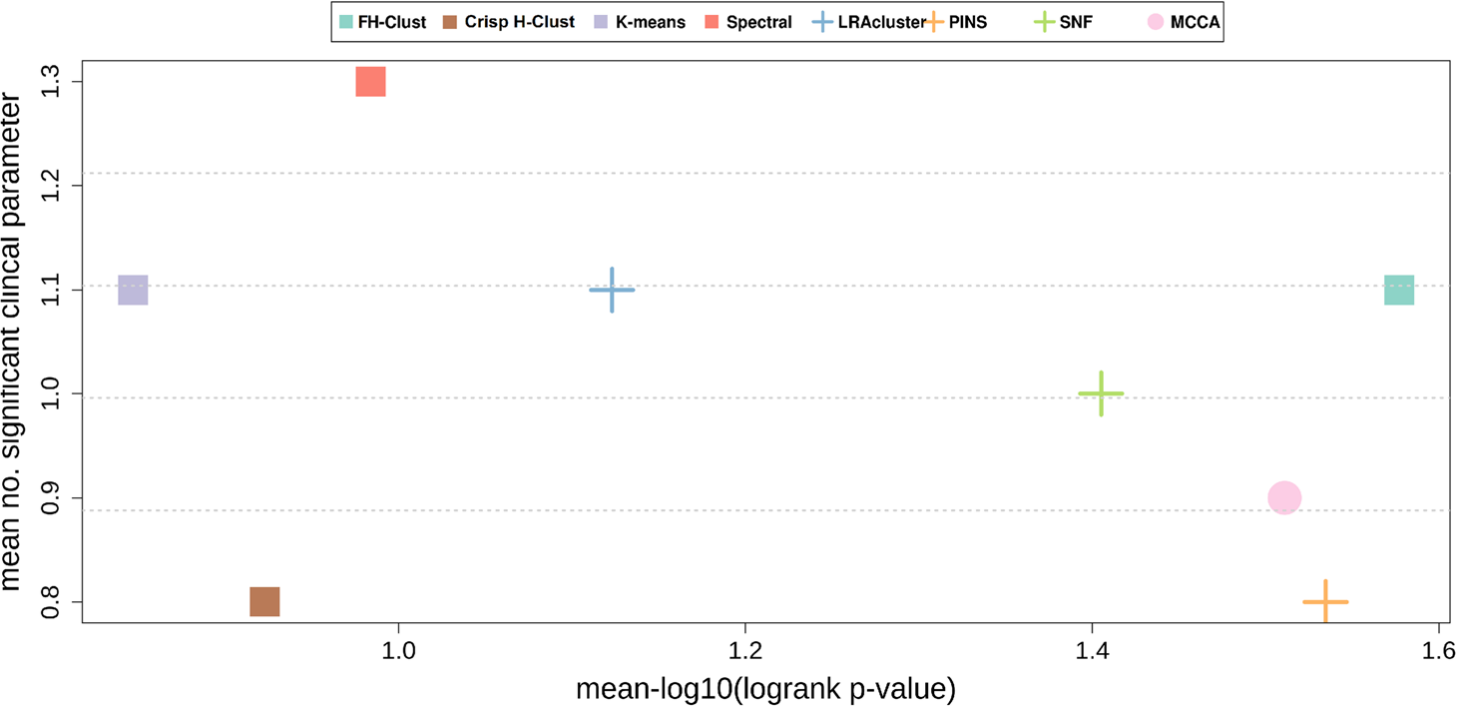
Mean performance of the algorithms on ten multi-omics cancer datasets. The x-axis measures the differential survival between clusters (mean -log10 of logrank’s test *p*-value), and the y-axis is the mean number of clinical parameters enriched in the clusters

**Fig. 7:**
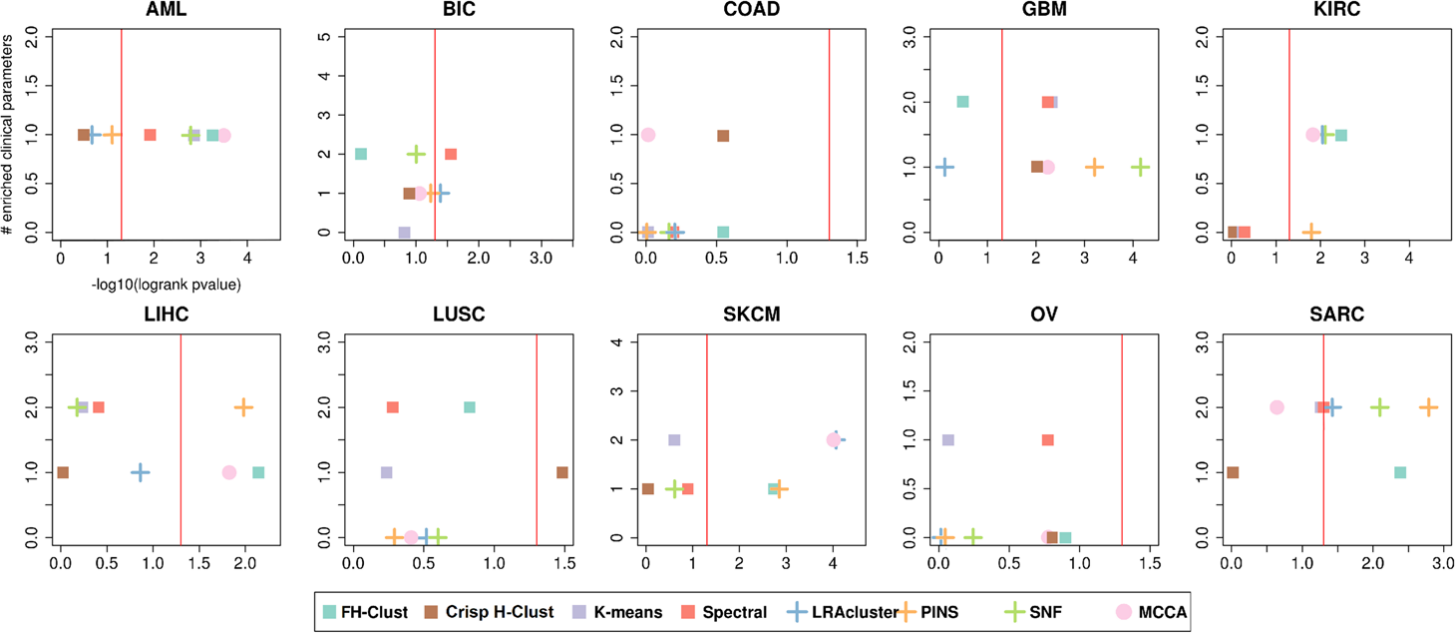
Performance of the algorithms on ten multi-omics cancer datasets. For each plot, the x-axis measures the differential survival between clusters(-log10 of logrank’s test *P*-value), and the y-axis is the number of clinical parameters enriched in the clusters. Red vertical lines indicate the threshold for significantly different survival (*P*-value ≤ 0.05)

Figure 6 shows the average performance for multi-omics data and for each single-omic separately, across all cancer types, and Fig. 7 shows the performance on the different cancer datasets. All algorithms show quite similar performance in either differential survival or enriched clinical parameters. With respect to survival, our *FH*-Clust method achieved the overall best prognostic value (*sum* of −*log*10*p*-values =15.77), while PINS (15.35) and MCCA (15.11) ranked, second and third, respectively.

In Table 2 the differential survival between clusters (mean −*log*10 of *logrank*’s test *p*-value) are reported. Spectral achieved the highest total number of significant clinical parameters, with 13 parameters. *FH*-Clust, along with LRAcluster and K-means placed themselves second with 11 parameters. SNF achieved the third position with 10 parameters.

**Table 2:** Performance on ten multi-omics: Number of clinical parameters enriched in the clusters

	AML	BIC	COAD	GBM	KIRC	LIHC	LUSC	SKCM	OV	SARC	Means
FH-Clust	1	2	0	2	1	1	2	1	0	1	1.1
Crisp H-Clust	1	1	1	1	0	1	1	1	0	1	0,8
K-means	1	0	0	2	0	2	1	2	1	2	1.1
Spectral	1	2	0	2	0	2	2	1	1	2	1.3
LRAcluster	1	1	0	1	2	1	0	2	0	2	1
PINS	1	1	0	1	0	2	0	1	0	2	0.8
SNF	1	2	0	1	1	2	0	1	0	2	1
MCCA	1	1	1	1	1	1	0	1	0	2	0.9

With respect to survival (Table 3), *FH*-Clust outperformed its competitors achieving 16 parameters. MCCA, PINS and SNF have achieved good results with 15, 15 and 14 enriched parameters, respectively.

**Table 3:** Performance on ten multi-omics: Differential survival between clusters (-log10 of logrank’s test *P*-value)

	AML	BIC	COAD	GBM	KIRC	LIHC	LUSC	SKCM	OV	SARC	Means
FH-Clust	3.24	0.18	0.61	0.49	2.16	2.08	0.81	2.83	0.89	2.42	1.57
Crisp H-Clust	0,55	0,72	0,51	1,96	0,06	0,06	1,48	0,12	0,74	0,06	0,65
K-means	2.92	0.62	0.01	2.32	0.15	0.23	0.23	0.6	0.06	1.29	0.84
Spectral	1.89	1.55	0.19	2.23	0.29	0.4	0.27	0.89	0.77	1.29	0.98
LRAcluster	0.68	1.38	0.22	0.12	2.04	0.72	0.52	4.08	0.05	1.42	1.12
PINS	1.14	1.23	0	3.2	1.79	1.98	0.29	2.85	0.04	2.78	1.53
SNF	2.87	1	0.16	4.15	2.12	0.17	0.6	0.61	0.24	2.09	1.4
MCCA	3.49	1.02	0.16	2.3	1.82	0.15	0.47	4.07	0.55	1.08	1.51

We also counted the number of datasets for which a method solution obtains significantly different survival. These results are reported in Table 4. All methods that were developed for multi-omics data had at least four cancer types with significantly different survival. In this case, *FH*-Clust and PINS had 5 different cancer subtypes for which its clustering had significantly different prognosis. *FH*-Clust, Spectral clustering and MCCA had enrichment in 8 cancer types.

**Table 4:** For each benchmarked algorithm, the number of cancer subtypes for which its clustering had significantly different prognosis (first row) and had at least one enriched clinical label (second row) are shown

	FH-Clust	K-means	Spectral	LRAcluster	PINS	SNF	MCCA
Significant different survival	5	2	3	4	5	4	4
Significant clinical enrichment	8	7	8	7	6	7	8

On average, *FH*-Clust, PINS and MCCA had better prognostic value, but found less enriched clinical labels as compared to spectral clustering method.

The number of clusters found for each dataset are presented in Table 5, ranging from 2 to 4. Because of the good methods performance in the previous analysis, partitioning the data into a relatively high number of clusters could indicate that clustering cancer patients into more clusters improves prognostic value and clinical significance.

**Table 5:** Number of clusters chosen by the benchmarked algorithms on ten multi-omics cancer datasets

	AML	BIC	COAD	GBM	KIRC	LIHC	LUSC	SKCM	OV	SARC	Means
FH-Clust	4	2	2	3	2	2	3	2	10	3	3,3
Crisp H-Clust	2	2	2	2	2	2	2	2	2	2	2
K-means	5	2	2	5	2	2	2	2	2	2	2,6
Spectral	9	3	2	5	2	2	2	2	4	2	3,3
LRAcluster	3	2	2	2	9	2	2	2	2	3	2,9
PINS	4	2	4	2	2	5	3	15	2	5	4,4
SNF	4	2	3	2	4	2	2	3	3	3	2,8
MCCA	3	2	7	2	3	2	2	6	2	2	3,1

**Fig. 8:**
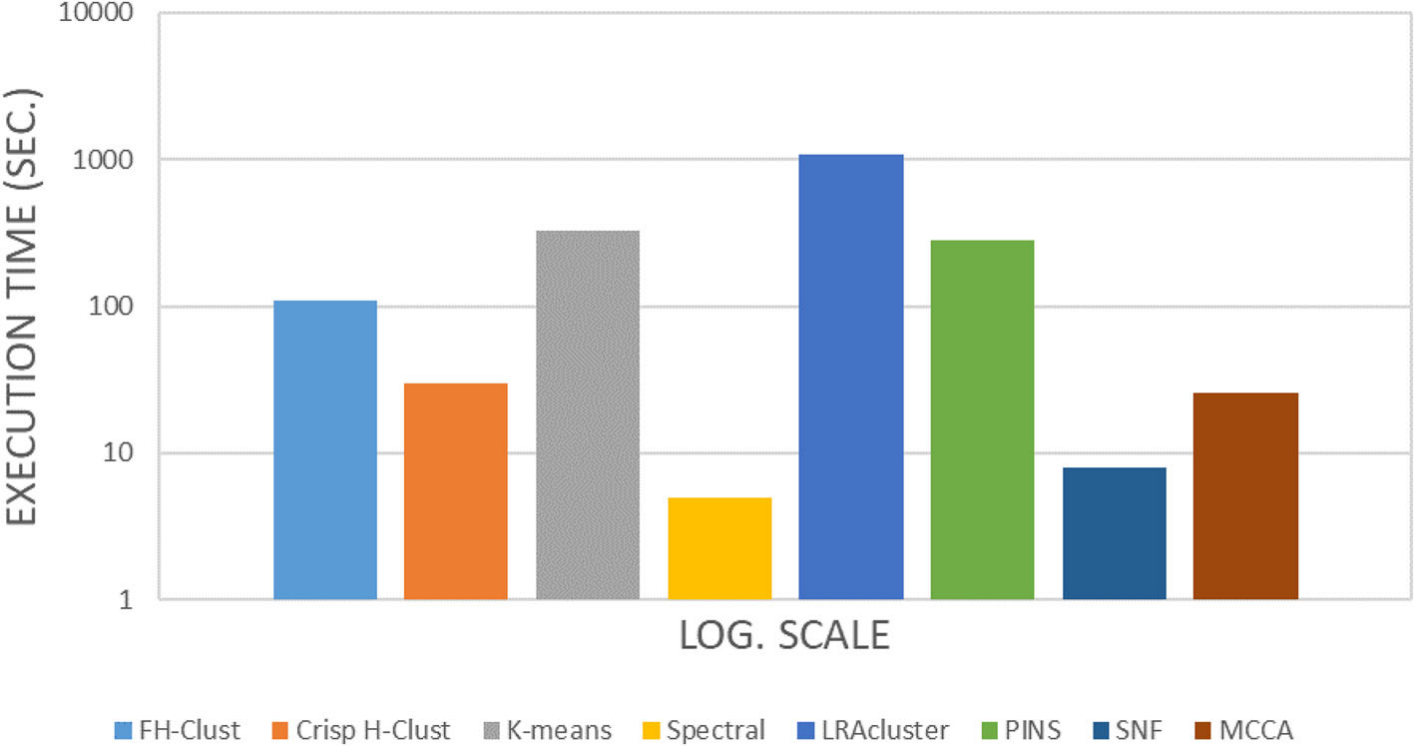
Computational time comparisons

Concerning with methods computational burden, their run times are reported in Table 6. *FH*-Clust takes, on average, 110 seconds per dataset, while Spectral and SNF got lower timing. The worst method takes roughly 18 minutes per dataset (see Fig. 8).

**Table 6:** Runtime in seconds of the algorithms on ten multi-omics cancer datasets

	AML	BIC	COAD	GBM	KIRC	LIHC	LUSC	SKCM	OV	SARC	Means
FH-Clust	21	460	40	59	32	123	94	167	58	49	110
Crisp H-Clust	17	70	20	22	19	35	30	32	25	27	30
K-means	97	748	160	197	108	342	389	736	322	194	329
Spectral	4	8	7	3	4	5	6	6	5	4	5
LRAcluster	390	3177	532	557	392	1268	1091	1761	780	771	1072
PINS	108	529	226	205	140	212	359	436	380	193	279
SNF	6	15	6	5	6	10	9	11	8	7	8
MCCA	15	53	16	17	16	34	29	31	23	21	26

**Fig. 9:**
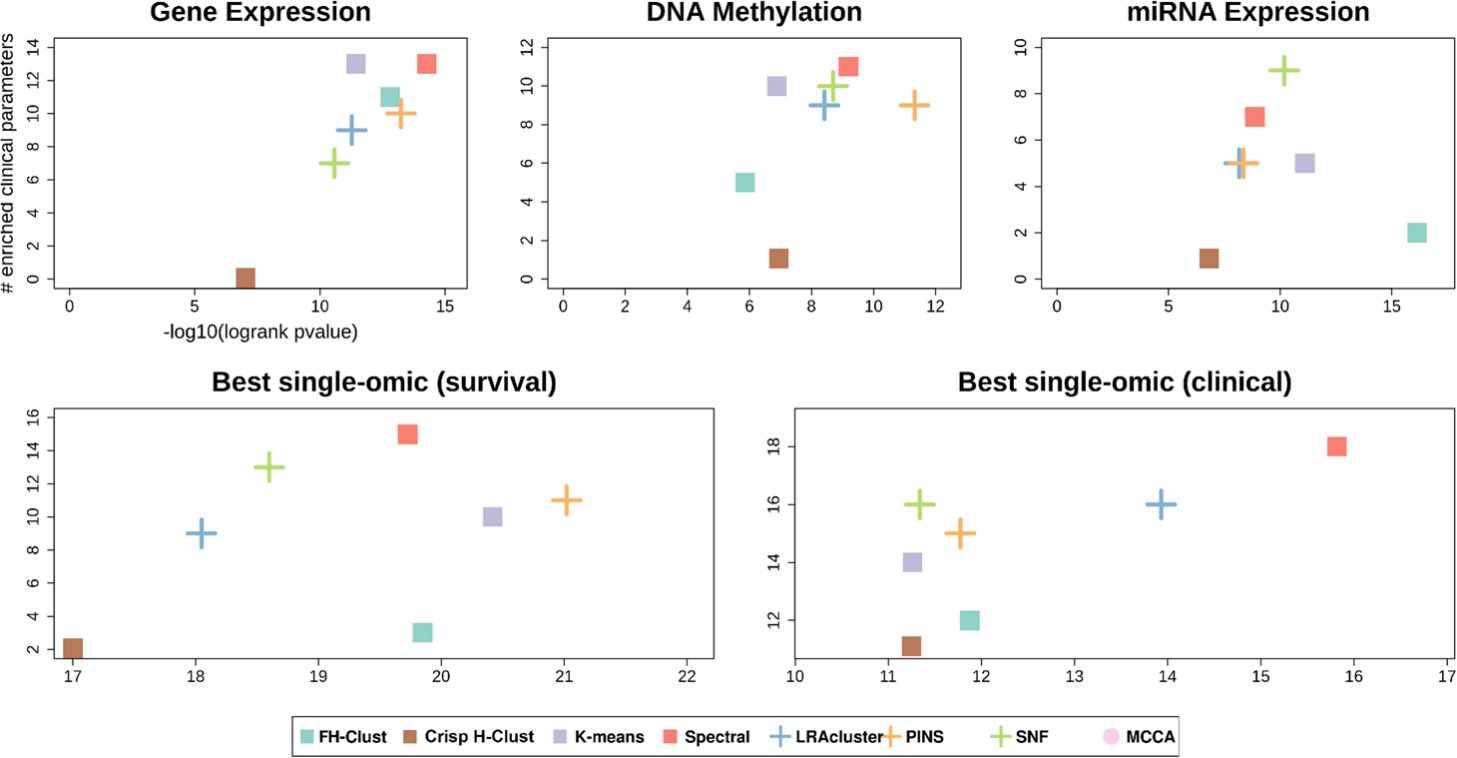
Summarized performance of the algorithms across ten cancer datasets. For each plot, the x-axis measures the total differential prognosis between clusters (sum across all datasets of –log10 of logrank’s test *P*-value), and the y-axis is the total number of clinical parameters enriched in the clusters across all cancer types. (**a**–**c**) Results for single-omic datasets. **d** Results when each method uses the single omic that achieves the highest significance in survival. **e** Same with respect to enrichment of clinical labels

Finally, Fig. 9 shows the benchmarked methods performance for single-omic data, moreover, for each dataset and method, the single omic that gave the best results for survival and clinical enrichment are also shown. These results suggest that *FH*-Clust provides better prognostic value and clinical significance on multi-omics data compared to the analysis of single-omic data used separately. Nevertheless, the interested reader may refer to the supplementary material for details on additional results concerning single-omics. We also stress that the proposed method, differently from other methods, such as SNF, does not need any hyperparameter tuning. Moreover, clustering is embedded in the data integration (and vice versa), and the use of fuzzy concepts (i.e., *t*-norms), from one hand, permits to obtain a generalisation of the clustering approaches whereas, on the other hand, gives the possibility to apply an inference system (e.g., Mamdani) for a quantitative and qualitative measure (e.g., “High”, “Medium”, “Low”) in cancer risk assessment.

## Conclusions

In this work, we proposed a multi-view clustering methodology for identifying patient subgroups from different *omics* data. In biological and biomedical fields, combining these omics data can significantly increase data mining capabilities. One of the main aspects of this methodology is the use of a measure of dissimilarity between sets of observations, by using an appropriate metric and a consensus matrix, that is a representative agglomerate information of all the dendrograms. As emerged from the analysis of the scientific literature, to the best of our knowledge our work concerns for the first time a model based on fuzzy logic used for the agglomeration of multi-omic data. The use of fuzzy logic allows us to introduce more flexible data mining features also related to approximate reasoning. Several experiments and comparisons have been made on real data (e.g., Glioblastoma, Prostate Cancer) to assess the proposed methodology. The results suggest that *FH*-Clust provides better prognostic value and clinical significance compared to analysis of single-omic data alone. Fuzzy Logic concepts, and in particular membership functions, permits to develop a fuzzy inference model (i.e., Mamdani, Fuzzy Cognitive Maps) for easily obtaining a model for a quantitative and qualitative risk assessment of the cancer. The model, based on approximate reasoning, can be particularly useful for embedded devices.

In future work, it could be possible to improve results for multi-omics analysis, in a number of ways. For instance, more accurate feature selection[19] algorithms could be adopted for improving the overall performance. On one hand, the integration of labelled data could improve the feature selection step. On the other hand, some specific feature extraction strategies could be adopted, indeed approaches based on the signal analysis of gene expression data (e.g., non-linear Principal Component Analysis, Compressive Sensing), could possibly further improve the performance [20, 21]. In future, it is possible to foresee a different weight for each omic data, in order to obtain a more robust similarity, and parametric similarity measures can be adopted (e.g., uninorm) for generalizing the concept of AND and OR connections between clusters.

## Supplementary information

**Additional file 1** Supplementary Material.

## Data Availability

Code and data of the proposed approach are available on: Multi-Omics-Cancer-BenchmarkGitHubrepository .

## Abbreviations

FH-Clust: Fuzzy-hierarchical CLUSTering
DNA: DeoxyriboNucleic acid
RNA: RiboNucleic acid
Hierarchical Clustering: HC
Crisp HC: Crisp hierarchical clustering
FL: Fuzzy logic
TCGA: The cancer genome atlas
AML: Acute myeloid leukemia
BIC: Breast invasive carcinoma
COAD: Colon adenocarcinoma
GBM: Glioblastoma multiforme
KIRC: Kidney renal clear cell carcinoma
LIHC: Liver hepatocellular carcinoma
LUSC: Lung squamous cell carcinoma
SKCM: Skim Cutaneous Melanoma
OV: Ovarian serous cystadenocarcinoma
SARC: Sarcoma
PINS: Perturbation Clustering for data INtegration and disease Subtyping
LRACluster: Low rank approximation based multi-omics data clustering
SNF: Similarity Network Fusion
MCCA: Multi Canonical Correlation Analysis

## Footnotes

1 Row data are available at: http://acgt.cs.tau.ac.il/multi_omic_benchmark/download.html

2 https://github.com/Shamir-Lab/Multi-Omics-Cancer-Benchmark/.
