# Supplementary material for: Data integration by fuzzy similarity-based hierarchical clustering
Source: BMC Bioinformatics. 2020 Aug 21;21(Suppl 10):350. doi: 10.1186/s12859-020-03567-6 (PMC7446192; doi:10.1186/s12859-020-03567-6)
Supplement: Supplementary file 1 — Additional file 1 Supplementary Material. [file 12859_2020_3567_MOESM1_ESM.pdf]

RESEARCH

# Data Integration by Fuzzy Similarity-Based Hierarchical Clustering Supplementary Material

Angelo Ciaramella[1*], Davide Nardone[2] and Antonino Staiano[1]

*Correspondence: angelo.ciaramella@uniparthenope.it
[1]Dipartimento di Scienze e Tecnologie, Università degli Studi di Napoli "Parthenope", Centro Direzionale, C4 Island, 80143 Naples, Italy
Full list of author information is available at the end of the article

## Abstract

**Background:** High throughput methods, in biological and biomedical fields, acquire a large number of molecular parameters or omics data by a single experiment. Combining these omics data can significantly increase the capability for recovering fine-tuned structures or reducing the effects of experimental and biological noise in data.

**Results:** In this work we propose a multi-view integration methodology (named $FH$-Clust) for identifying patient subgroups from different *omics* information (e.g., *Gene Expression*, *Mirna Expression*, *Methylation*). In particular, hierarchical structures of patient data are obtained in each omic (or view) and finally their topologies are merged by consensus matrix. One of the main aspects of this methodology, is the use of a measure of dissimilarity between sets of observations, by using an appropriate metric. For each view, a dendrogram is obtained by using a hierarchical clustering based on a fuzzy equivalence relation with *Łukasiewicz* valued fuzzy similarity. Finally, a consensus matrix, that is a representative information of all dendrograms, is formed by combining multiple hierarchical agglomerations by an approach based on transitive consensus matrix construction. Several experiments and comparisons are made on real data (e.g., Glioblastoma, Prostate Cancer) to assess the proposed approach.

**Conclusions:** Fuzzy logic allows us to introduce more flexible data agglomeration techniques. From the analysis of scientific literature, it appears to be the first time that a model based on fuzzy logic is used for the agglomeration of multi-omic data. The results suggest that $FH$-Clust provides better prognostic value and clinical significance compared to the analysis of single-omic data alone and it is very competitive with respect to other techniques from literature.

**Keywords:** Multi-omics data; Data Integration; Hierarchical Clustering; Fuzzy Similarity; Fuzzy Aggregation

## 1 Membership functions

Fuzzification is the process of converting a crisp input value to a fuzzy value. Many types of curves and tables can be used, for example triangular or trapezoidal-shaped membership functions are easier to represent in embedded controllers. In this work, fuzzy sets are represented by [1]

$$\mu(\mathbf{x}_i) = \frac{\mathbf{x}_i - \min(\mathbf{x}_i)}{\max(\mathbf{x}_i) - \min(\mathbf{x}_i)}, \tag{1}$$

where $\mathbf{x}_i = [x_1^i, x_2^i, \ldots, x_L^i]$ is the $i$-the observation vector of $L$ features.

## 2 Fuzzy similarity

Fuzzy sets can be combined via the conjunction and disjunction operations (continuous triangle norms or co-norms, respectively). A *triangular norm* (*t-norm* for short), is a binary operation $t : [0,1]^2 \to [0,1]$ satisfying, for all $x, y, z \in [0,1]$, the axioms of [2]

$$\begin{array}{lcll} t(x,y) & = & t(y,x) & (\textit{commutativity}) \\ t(x,t(y,z)) & = & t(t(x,y),z) & (\textit{associativity}) \\ t(x,y) & \leq & t(x,z) \quad \text{whenever } y \leq z & (\textit{monotonicity}) \\ t(x,1) & = & x & (\textit{boundary condition}) \end{array} \tag{2}$$

The most used $t$-norms, in practical applications, are [3]

$$\begin{array}{lcll} t_{\mathbf{M}}(x,y) & = & \min(x,y) & (\textit{minimum}) \\ t_{\mathbf{P}}(x,y) & = & x \cdot y & (\textit{product}) \\ t_{\mathbf{L}}(x,y) & = & \max(x+y-1,0) & (\textit{Łukasiewicz } t\textit{-norm}) \\ t_{\mathbf{D}}(x,y) & = & \begin{cases} 0 & \text{if } (x,y) \in [0,1]^2 \\ \min(x,y) & \text{otherwise} \end{cases} & (\textit{drastic product}) \end{array} \tag{3}$$

In order to introduce the fuzzy similarity, in the following, we focus on the properties of the Łukasiewicz $t$-norm ($t_{\mathbf{L}}$). One main operator adopted in fuzzy-based systems is the *residuum* $\to_t$

$$x \to_t y = \bigvee \{z | t(z,x) \leq y\}, \tag{4}$$

where $\bigvee$ is the *union* operator and, for the left-continuous basic $t$-norm $t_{\mathbf{L}}$, is given by

$$x \to_{\mathbf{L}} y \quad = \quad \min(1-x+y, 1) \quad (\textit{Łukasiewicz implication}). \tag{5}$$

Consequently, on a residuated lattice, the *bi-residuum* can defined as

$$x \leftrightarrow_t y = (x \rightarrow_t y) \wedge (y \rightarrow_t x), \tag{6}$$

where $\wedge$ is the *meet*. It is worth noting that with $t_{\mathbf{L}}$ we obtain the following *bi-residuum*

$$x \leftrightarrow_{\mathbf{L}} y \quad = \quad 1 - \max(x, y) + \min(x, y). \tag{7}$$

On the other hand, a binary *fuzzy relation* $R$ is defined on $U \times V$ as a fuzzy set on $U \times V$ ($R \subseteq U \times V$). A *similarity matrix* is a particular fuzzy relation $S \subseteq U \times U$ such that, for each $u, v, w \in U$, the following properties are satisfied

$$\begin{array}{lcll} S\langle u,u\rangle & = & 1 & (\textit{everthing is similar to itself}) \\ S\langle u,v\rangle & = & S\langle v,u\rangle & (\textit{symmetric}) \\ t(S\langle u,v\rangle, S\langle v,w\rangle) & \leq & S\langle u,w\rangle & (\textit{weakly transitive}). \end{array} \tag{8}$$

It is essential to observe that, from fuzzy sets with membership functions $\mu : X \rightarrow [0, 1]$, a fuzzy similarity matrix $S$ can be generated as

$$S\langle a, b\rangle = \mu(a) \leftrightarrow_t \mu(b), \tag{9}$$

for all $a, b \in X$.

Now, to build a fuzzy similarity matrix by considering all features of a dataset, a main result must be considered [1, 2]:

**Proposition 1** *Consider $n$ Łukasiewicz valued fuzzy similarities $S_i$, $i = 1, \ldots, n$ on a set $X$. Then*

$$S\langle x, y\rangle = \frac{1}{n} \sum_{i=1}^{n} S_i\langle x, y\rangle \tag{10}$$

*is a Łukasiewicz valued fuzzy similarity on $X$.*

In this work, we consider for equation 10

$$S_i\langle x, y\rangle = x \leftrightarrow_{\mathbf{L}} y. \tag{11}$$

Letting $t_{\mathbf{L}}$ be the Łukasiewicz product, it is worth noting that $S$ is a fuzzy equivalence relation on $X$ with respect to (w.r.t.) $t_{\mathbf{L}}$ iif $1 - S$ is a *pseudo-metric* on $X$.

## 3 Dendrogram and Consensus Matrix

If a similarity relation is *min-transitive* ($t$ = min in properties (8)) then it is a *fuzzy-equivalence relation* that can be graphically described by a *dendrogram* [4]. In other words, transitivity implies the existence of the dendrogram.
The min-transitive closure $R^T$ of $R$ can be obtained as follows [5]

$$R^T = \bigcup_{i=1}^{n-1} R^i \tag{12}$$

where $R^{i+1}$ is defined as

$$R^{i+1} = R^i \circ R, \tag{13}$$

and $n$ is the dimension of a relation matrix.
Considering two fuzzy relations $R$ and $S$, the composition $R \circ S$ is a fuzzy relation defined by

$$R \circ S\langle x, y\rangle = \mathrm{Sup}_{z \in X}\{R\langle x, z\rangle \odot S\langle z, y\rangle\} \tag{14}$$

$\forall x, y \in X$, where $\odot$ stands for a $t$-norm (e.g., min operator) [5]. Thus, the min-transitive closure $R^T$ of a matrix $R$ can be easily computed and the overall process is described in Algorithm 1.

1: **Input:** relation $R$
2: **Output:** transitive relation $R^T$
3: **Elaborate:**
1. Compute $R^* = R \cup (R \circ R)$
2. if $R^* \neq R$ replace $R$ with $R^*$ and go to step 1
else $R^T = R^*$ and the algorithm terminates.

**Algorithm 1:** : Min-transitive closure

After the dendrograms have been obtained each time, a consensus matrix, i.e., the representative information of all dendrograms, is obtained by combining the transitive closures by using equation 14 (i.e., max-min) [5]. The overall approach is described in Algorithm 2.

1: **Input** $S^{(i)}$, $1 \leq i \leq L$ $L$ input similarity matrices (dendrograms)
2: **Output** similarity matrix (dendrogram) $S$
    1. Aggregate the similarity matrices to a final similarity matrix
    $S = Aggregate(S^{(1)}, S^{(2)}, \ldots, S^{(L)})$
        a. Let $S^*$ be the identity matrix
        b. For each $S^{(i)}$ calculate e $S^* = S^* \cup (S^* \circ S^{(i)})$
        c. If $S^*$ is not changed $S = S^*$ and goto step 3 else goto step 1.b
3: Create the final dendrogram from $S$

**Algorithm 2:** : Combination of dendrograms

**Author details**
[1]Dipartimento di Scienze e Tecnologie, Università degli Studi di Napoli “Parthenope”, Centro Direzionale, C4 Island, 80143 Naples, Italy. [2]Hitachi Rail STS, Via Argine, 425, 80147 Naples, Italy.

**References**

1. Turunen, E.: Mathematics Behind Fuzzy Logic. Advances in Soft Computing, Springer-Verlag, ??? (1999)
2. Sessa, S., Tagliaferri, R., Longo, G., Ciaramella, A., A., S.: Fuzzy similarities in stars/galaxies classification. Proceedings of IEEE International Conference on Systems, Man and Cybernetics, 494–4962 (2003)
3. Ciaramella, A., Tagliaferri, R., Pedrycz, W.: The genetic development of ordinal sums. Fuzzy Sets and Systems **151**(2), 303–325 (2005)
4. Meyer, H.D., Naessens, H., Baets, B.D.: Algorithms for computing the min-transitive closure and associated partition tree of a symmetric fuzzy relation. Eur. Journal Oper. Res. **155 (1)**, 226–238 (2004)
5. Mirzaei, A., Rahmati, M.: Hierarchical-clustering-combination scheme based on fuzzy-similarity relations. IEEE Transaction on Fuzzy Systems **18 (1)**, 27–39 (2010)
